# Phosphorus Doping Strategy-Induced Synergistic Modification of Interlayer Structure and Chemical State in Ti_3_C_2_T_x_ toward Enhancing Capacitance

**DOI:** 10.3390/molecules28134892

**Published:** 2023-06-21

**Authors:** Lihong Chen, Yifan Bi, Yunqi Jing, Jun Dai, Zhenjiang Li, Changlong Sun, Alan Meng, Haijiao Xie, Minmin Hu

**Affiliations:** 1 School of Materials Science and Engineering, Qingdao University of Science and Technology, Qingdao 266042, China; 2 College of Environment and Safety Engineering, Qingdao University of Science and Technology, Qingdao 266042, China; 3 College of Electromechanical Engineering, Qingdao University of Science and Technology, Qingdao 266061, China; 4 State Key Laboratory Base of Eco-Chemical Engineering, College of Chemistry and Molecular Engineering, Qingdao University of Science and Technology, Qingdao 266042, China; 5 Hangzhou Yanqu Information Technology Co., Ltd., Hangzhou 310003, China

**Keywords:** MXene, supercapacitor, energy storage, pseudocapacitance, P doping

## Abstract

Heteroatom doping is considered an effective method to substantially improve the electrochemical performance of Ti_3_C_2_T_x_ MXene for supercapacitors. Herein, a facile and controllable strategy, which combines heat treatment with phosphorous (P) doping by using sodium phosphinate (NaH_2_PO_2_) as a phosphorus source, is used to modify Ti_3_C_2_T_x_. The intercalated ions from NaH_2_PO_2_ act as “pillars” to expand the interlayer space of MXene, which is conducive to electrolyte ion diffusion. On the other hand, P doping tailors the surface electronic state of MXene, optimizing electronic conductivity and reducing the free energy of H^+^ diffusion on the MXene surface. Meanwhile, P sites with lower electronegativity owning good electron donor characteristics are easy to share electrons with H^+^, which is beneficial to charge storage. Moreover, the adopted heat treatment replaces –F terminations with O-containing groups, which enhances the hydrophilicity and provides sufficient active sites. The change in surface functional groups increases the content of high valence-stated Ti with a high electrochemical activity that can accommodate more electrons during discharge. Synergistic modification of interlayer structure and chemical state improves the possibility of Ti_3_C_2_T_x_ for accommodating more H^+^ ions. Consequently, the modified electrode delivers a specific capacitance of 510 F g^−1^ at 2 mV s^−1^, and a capacitance retention of 90.2% at 20 A g^−1^ after 10,000 cycles. The work provides a coordinated strategy for the rational design of high-capacitance Ti_3_C_2_T_x_ MXene electrodes.

## 1. Introduction

Electrochemical energy storage devices are considered one of the most promising energy storage technologies to solve the global energy crisis, with batteries and supercapacitors as the two main devices [1,2]. In particular, supercapacitors have significant advantages such as high specific energy, high power density, long cycle life, and rapid charging and discharge [3,4,5], which can be classified into electrical double layer capacitors (EDLCs) and pseudocapacitors (PCs) [6]. EDLCs tend to exhibit lower energy density due to the limited accessible surface area, instead, PCs can store more charges by their Faradaic redox mechanism [7,8]. However, most well-studied pseudocapacitive materials, such as conducting polymers and transition metal oxides, suffer from volumetric swelling/contraction, limited cycling performance, and high electrode resistance [9,10]. Thus, the exploration of new electrode materials with outstanding electrochemical performance is an imminent challenge for developing high-performance energy storage devices.

MXene, a family of two-dimensional (2D) transition metal carbide/nitride, has attracted great attention as a pseudocapacitive electrode material in electrochemical energy storage due to its good conductivity, excellent hydrophilicity, large specific surface area, high mechanical strength, and adjustable interlayer spacing [11,12,13,14,15,16,17]. The most promising type of MXene, Ti_3_C_2_T_x_, is obtained by etching Al layers in the Ti_3_AlC_2_ MAX phase with HF [18,19,20], HCl–LiF [21], or Lewis acidic molten salt [22], where T represents terminal groups –O, –OH, or –F on the surface of Ti_3_C_2_T_x_, and x is the number of terminal groups [23,24]. In H_2_SO_4_ electrolyte, Ti_3_C_2_T_x_ has a high theoretical capacitance due to the efficient electric double layer (EDL) charge accumulation in the interlayer space and the fast pseudocapacitive proton insertion/desertion by surface redox reactions [25].

Generally, the capacitive performance of MXene is primarily determined by its surface chemistry state, which dictates the interaction mechanism and binding strength between the adsorbates and MXenes and thus contributes to the electrochemical performance [26]. Among the terminations of MXene (–O, –OH, –F), –O groups with lower electronegativity (3.5) own good electron donor characteristics, which not only facilitates the electrostatic accumulation of electrons at the electrode surface, giving rise to the increase in EDL capacitance but also works as active sites for proton bonding/debonding, leading to the enhancement in pseudocapacitance. Moreover, when MXene is doped with heteroatom N with lower electronegativity (3.04), a significant improvement in electrochemical performance is achieved by structural, compositional, and surface electronic regulation. For example, Yang et al. prepared nitrogen-doped Ti_3_C_2_T_x_ by annealing in ammonia, which yields 192 F g^−1^ at 1 mV s^−1^ in 1 M H_2_SO_4_ and remains 67% of initial capacitance over the scan rates from 1 to 200 mV s^−1^ [27]. Zhang et al. prepared a nitrogen-doped Ti_3_C_2_T_x_ film electrode with a hydrothermal method using hydrazine hydrate as a nitrogen source, which delivers 340 F g^−1^ at 2 mV s^−1^ and no capacitance degradation after 10,000 cycles in 1 M H_2_SO_4_ [28]. Pu et al. prepared a new type of N-doped MXene electrode from non-toxic biological chitosan, which delivers 286.28 F g^−1^ and maintains almost 100% retention after 10,000 cycles [29]. Therefore, we believe that introducing phosphorus heteroatom with lower electronegativity (2.19) can develop a higher capacitance MXene electrode by trimming the electron donor capability, and modifying the surface electrical and chemical properties. In addition, P heteroatom doping is considered an effective and convenient modification strategy to improve the electrochemical performance of carbon materials [30,31]. Although there are several reported works in regard to P doping MXene in supercapacitors, the doping effect is not ideal and the underlying doping mechanism, especially the doping positions/configurations, and their effect on capacitance enhancement are still unexplored.

Inspired by the above motivation, in this work, a phosphorus doping strategy (high-temperature heat treatment with NaH_2_PO_2_ as the phosphorus source) was employed to obtain the electrode material of supercapacitor (P–Ti_3_C_2_T_x_) with high specific capacitance and excellent cyclic performance. Morphology and component characterization, DFT calculation, and electrochemical kinetic analysis have been used to give deep insight into the mechanism of improvement in electrochemical performance. As revealed, P doping improved electrical conductivity and reduced the free energy of H^+^ diffusion on the MXene surface. Meanwhile, the intercalated ions acted as “pillars” to expand the interlayer space of MXene, which is conducive to electrolyte ion diffusion. Moreover, the adopted high-temperature heat treatment in the P doping strategy replaced –F terminations with O-containing groups, which enhances the hydrophilicity and provides sufficient electrochemical active sites for H^+^. Synergistic modification of interlayer structure and chemical state improves the possibility of Ti_3_C_2_T_x_ for accommodating more H^+^ ions, further enhancing the capacitance of Ti_3_C_2_T_x_.

## 2. Results and Discussion

### 2.1. Materials Characterization

**Figure 1:**
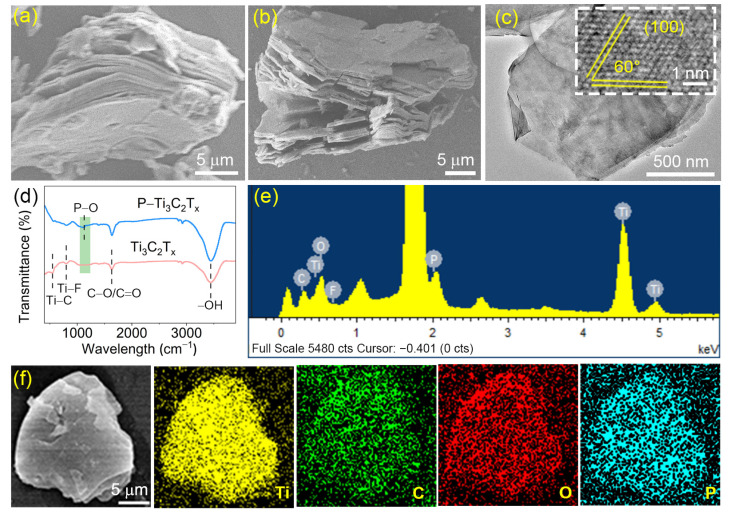
Morphology and structure characterization of Ti_3_C_2_T_x_ and P–Ti_3_C_2_T_x_. (**a**) SEM of Ti_3_C_2_T_x_; (**b**) SEM and (**c**) TEM images of P–Ti_3_C_2_T_x_ (inset shows the HRTEM image along the [0001] zone axis); (**d**) FTIR spectra of Ti_3_C_2_T_x_ and P–Ti_3_C_2_T_x_; (**e**,**f**) EDS spectrum and element mapping images of P–Ti_3_C_2_T_x_.

The morphologies and microstructures of the prepared samples were analyzed by using scanning electron microscopy (SEM) and transmission electron microscopy (TEM). Ti_3_C_2_T_x_ and P–Ti_3_C_2_T_x_ display the same structure with an accordion shape (Figure 1a,b), proving that the surface morphology of Ti_3_C_2_T_x_ is not affected after annealing treatment with NaH_2_PO_2_·H_2_O. The smooth and thin P–Ti_3_C_2_T_x_ nanosheets are observed in the TEM image (Figure 1c), further indicating the maintenance of a lamellar structure. Moreover, a high-resolution transmittance electron microscopy (HRTEM) image of P–Ti_3_C_2_T_x_ (Figure 1c insert) shows a hexagonal structure demonstrated by different grain orientations with 60° angles and lattice fringes of 0.26 nm which is attributed to the (100) lattice planes of the Ti_3_C_2_T_x_. Fourier transform infrared (FTIR) spectra were used to investigate the change in the surface chemical state of Ti_3_C_2_T_x_ upon P doping (Figure 1d), which indicates the presence of several common peaks belonging to Ti_3_C_2_T_x_ MXene, such as Ti–C (~555 cm^−1^), Ti–F (~800 cm^−1^), C–O/C=O (~1640 cm^−1^) and –OH (~3440 cm^−1^) in the range of 400–4000 cm^−1^ [32,33,34]. Notably, the new peak in P–Ti_3_C_2_T_x_ appearing near 1120 cm^−1^ corresponds to the P–O bond, which suggests that P atoms are successfully doped in Ti_3_C_2_T_x_ [26]. In addition, the energy dispersive spectroscopy (EDS) spectrum and element mapping images display the presence and uniform distribution of Ti, C, O, and P elements in P–Ti_3_C_2_T_x_ (Figure 1e,f), testifying that P atoms are successfully doped, which match well with the result of FTIR analysis.

**Figure 2:**
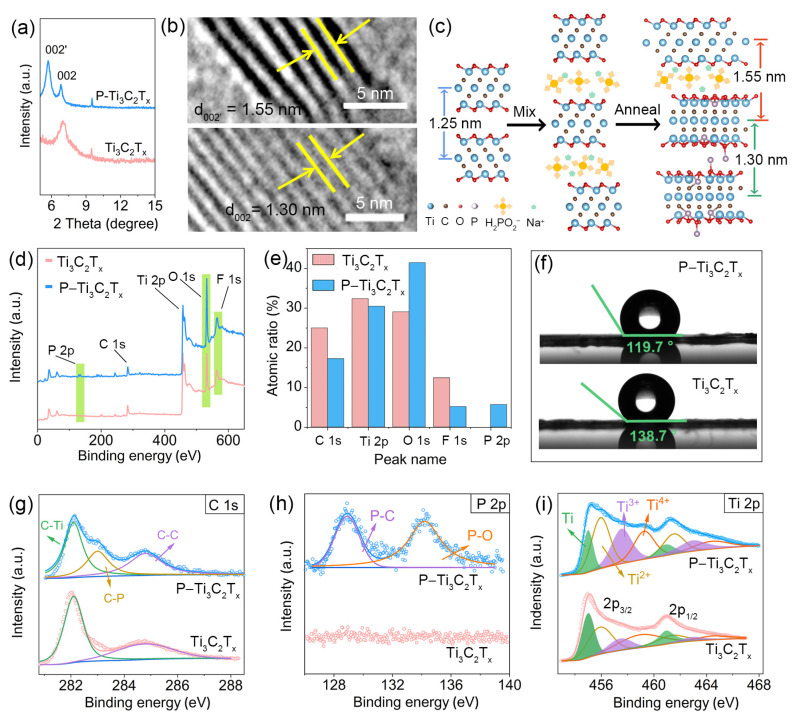
Characterization of the structure and chemical composition of Ti_3_C_2_T_x_ and P–Ti_3_C_2_T_x_. (**a**) XRD pattern of Ti_3_C_2_T_x_ and P–Ti_3_C_2_T_x_; (**b**) HRTEM images along the [11$\overset{–}{2}$0] zone axis of P–Ti_3_C_2_T_x_; (**c**) Structure model diagrams and corresponding schematic of the interlayer regulation in Ti_3_C_2_T_x_; (**d**,**e**) Full XPS spectrum and corresponding different element proportions of Ti_3_C_2_T_x_ and P–Ti_3_C_2_T_x_; (**f**) Contact angle of water on Ti_3_C_2_T_x_ and P–Ti_3_C_2_T_x_; The corresponding high-resolution XPS spectra of (**g**) C 1s, (**h**) P 2p, (**i**) Ti 2p.

To further investigate the lattice structure of MXene, X-ray diffraction (XRD) and HRTEM characterization was conducted. As shown in Figure 2a, the characteristic (002) peak of Ti_3_C_2_T_x_ shifted to a lower angle after the introduction of the P atom. According to the Bragg equation, the lattice spacing changes from 1.25 nm to 1.30 nm and 1.55 nm. The corresponding HRTEM images of P–Ti_3_C_2_T_x_ (Figure 2b) also exhibit characteristic lattice fringe spaces of 1.55 nm of (002′) and 1.30 nm of (002), which match well with the XRD results. The expansion of interlayer space should attribute to the successful insertion of Na^+^ and H_2_PO_2_^−^ in the layers of Ti_3_C_2_ during the ultrasonic treatment, which also leads to the P–Ti_3_C_2_T_x_ exhibiting a larger BET specific surface area than pristine Ti_3_C_2_T_x_ (Table S1). To exclude that the expansion of interlayer spacing is due to the annealing treatment, the XRD pattern of Ar–Ti_3_C_2_T_x_ under the same conditions without NaH_2_PO_2_·H_2_O was also conducted. As shown in Figure S3, the (002) peak of Ar–Ti_3_C_2_T_x_ moved to the right, indicating the expansion of interlayer spacing is not caused by heat treatment. The larger interlayer spacing and specific surface area favor undoubtedly the rapid diffusion of electrolyte ions and provide enough space to accommodate more electrolyte ions. Moreover, we noted that the characteristic peaks of TiO_2_ (PDF# 21-1272) appear in Ar–Ti_3_C_2_T_x_, and the TiO_2_ with lower conductivity losses partial pseudocapacitance of the Ti_3_C_2_T_x_ (Figure S3). Fortunately, due to the reductive nature of NaH_2_PO_2_, MXene is not oxidized in the P doping process. In addition, the (002) peak of P–Ti_3_C_2_T_x_ also splits into two peaks (002 and 002′) as shown in Figure 2a. Here, we presume that the split-up of the (002) peak in P–Ti_3_C_2_T_x_ is due to the partial decomposition of the intercalated H_2_PO_2_^−^ during annealing. Na^+^ and H_2_PO_2_^−^ ions enter MXene interlayers in the ultrasonic process, expanding the interlayer spacing. Then, the partial decomposition of H_2_PO_2_^−^ during annealing makes the enlarged interlayer spacing become smaller. Even so, fortunately, P–Ti_3_C_2_T_x_ still keeps larger interlayer spacing compared with that of pristine Ti_3_C_2_T_x_. Figure 2c illustrates the structure evolution of Ti_3_C_2_T_x_ during the preparation of P–Ti_3_C_2_T_x_ and the phenomenon of (002) peak splitting.

X-ray photoelectron spectroscopy (XPS) studies were then initiated to further investigate the change in component and valence states of Ti_3_C_2_T_x_ MXene on P doping. The full XPS spectra of P–Ti_3_C_2_T_x_ and Ti_3_C_2_T_x_ shown in Figure 2d reveal that they all possess Ti, C, O, and F elements. Notably, the P peak appears in P–Ti_3_C_2_T_x_, which proves the successful introduction of P atoms. The P atom is less electronegative and easily shares electrons with electrolyte ions (H^+^), which is beneficial to charge storage. Moreover, the intensity of the F 1s peak decreased whereas the intensity of the O 1s peak increased after doping, suggesting that part of the fluorine group was detached from the surface of Ti_3_C_2_T_x_ and replaced with oxygen-containing functional groups during the annealing treatment. This is consistent with the previous study that –F is unstable and –O is more stable [35]. The detailed change in atomic ratio of various elements in MXene is displayed in Figure 2e where after implementing the P doping strategy, the O atom content increased from 29.05% to 41.47%, and the F atom content decreased from 12.56% to 5.47%. To further confirm the variation in the surface functional groups of MXene, the surface contact angles of water on P–Ti_3_C_2_T_x_ and pristine Ti_3_C_2_T_x_ were tested, respectively. As shown in Figure 2f, the contact angle of water on P–Ti_3_C_2_T_x_ considerably decreased due to the enhancement of the content of O-containing groups, which increases the hydrophilicity of MXene and is conducive to the contact between active sites and electrolyte ions, thus enhancing the electrochemical performance of the electrode. Based on the previously reported electrochemical reaction occurring in Ti_3_C_2_T_x_ MXene [26]: ${\mathrm{Ti}}_{3}C_{2}O_{x}{\left(\mathrm{OH}\right)}_{y}F_{z}+{\;\delta H}^{+}+{\delta e}^{+}↔{\mathrm{Ti}}_{3}C_{2}O_{x-\delta }{\left(\mathrm{OH}\right)}_{y+\delta }F_{z}$, we suppose that the reduction in the number of –F functional groups and the increase in O-containing groups would greatly improve the capacitance [4].

High-resolution XPS can better analyze the change in the valence state of various atoms. From high-resolution XPS spectra of C 1s (Figure 2g), we can see that the Ti_3_C_2_T_x_ shows two peaks around 282.1 eV and 284.8 eV corresponding to C–Ti and C–C [36], respectively. Interestingly, P–Ti_3_C_2_T_x_ displays an additional C–P bond at 283.0 eV, which possibly is caused by P doping to the exposed edge of Ti_3_C_2_T_x_ [37]. The P 2p spectrum is further studied to analyze the mechanism of P doping (Figure 2h). It can be observed that there is no P peak in the pristine Ti_3_C_2_T_x_, while in the P–Ti_3_C_2_T_x_ sample, two peaks appear. By Gaussian fitting, the P–C bond and P–O bond can be corresponding at 128.9 eV and 134.2 eV, respectively [26,37]. It means the P dopant can be attached to the surface of Ti_3_C_2_T_x_ by adsorption/substitution. The presence of P–O groups would optimize the redox reaction process due to the lower electronegativity of the P atom. Moreover, the Ti 2p spectra obtained by Gaussian fitting are shown in Figure 2i. The Ti 2p_3/2_ spectra can be deconvoluted into four configurations, in which the binding energies are assigned to Ti (~455 eV), Ti^2+^ (~456 eV), Ti^3+^ (~457.6 eV), and Ti^4+^ (~459.3 eV) [36]. Furthermore, we can see that the content of the +3 valence state in P–Ti_3_C_2_T_x_ (28.4%) is higher compared with Ti_3_C_2_T_x_ (13.4%). The increase in valence may be due to the transition of the terminal group –F to O-containing groups on the surface of Ti_3_C_2_T_x_ and the introduction of P during annealing treatment. The higher valence can hold more electrons during the discharge process, thus increasing its specific capacitance [8].

### 2.2. Density Functional Theory Calculations

**Figure 3:**
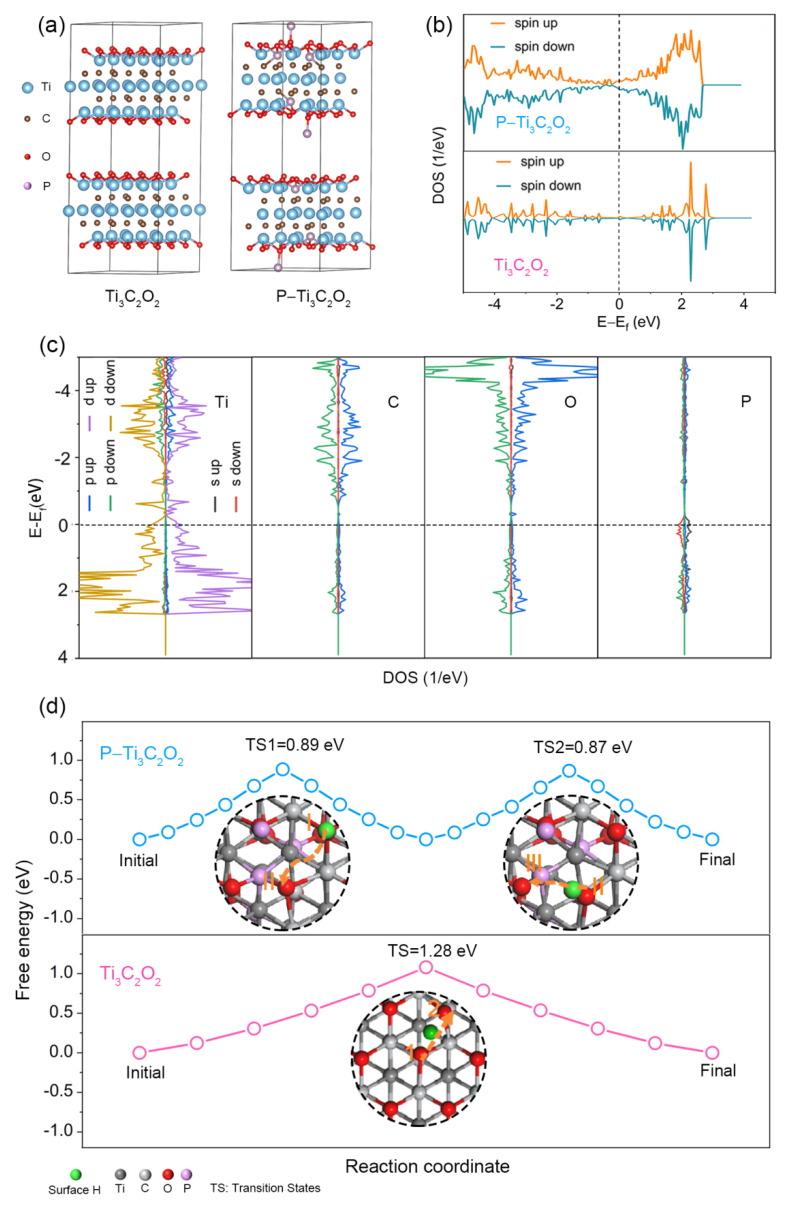
Calculation results based on DFT for P–Ti_3_C_2_O_2_ and Ti_3_C_2_O_2_. (**a**) Model structure of Ti_3_C_2_O_2_ and P–Ti_3_C_2_O_2_; (**b**) TDOS of P–Ti_3_C_2_O_2_ and Ti_3_C_2_O_2_; (**c**) PDOS of P–Ti_3_C_2_O_2_; (**d**) Diffusion free energy of H diffusion along different paths on the P–Ti_3_C_2_O_2_ and Ti_3_C_2_O_2_ surfaces.

To better understand the effect of P doping on the properties of MXene, we conducted theoretical calculations of the electronic structure before and after P doping. A supercell of Ti_3_C_2_O_2_ was constructed as the initial calculated model because O is the dominant functional group after annealing treatment (Figure 3a). Furthermore, according to the atomic ratio of P/Ti in the XPS result and the proportion of P–O and P–C bonds in P 2p spectra (Figure 2e,h), five Ti atoms are substituted by five P atoms, and the other three P atoms are bonded with O atoms directly in the supercell to approximate the P atoms concentration in P–Ti_3_C_2_T_x_. The constructed structure denoted as P–Ti_3_C_2_O_2_ is shown in Figure 3a. Figure 3b displays the total density of states (TDOS) of P–Ti_3_C_2_O_2_ and Ti_3_C_2_O_2_, respectively. The modulation of electronic configuration and spin polarization leads to the variation of DOS curves of P–Ti_3_C_2_O_2_ for both the up-spin and down-spin states. Both Ti_3_C_2_O_2_ and P–Ti_3_C_2_O_2_ display non-zero peaks across the Fermi level, which implies they possess zero band gaps. Notably, the P–Ti_3_C_2_O_2_ has higher DOS whether at the valence band (VB) or at the conduction band (CB) across the Fermi level, manifesting that electrons could easily migrate from VB to CB, implying the enhanced electronic conductivity of P–Ti_3_C_2_O_2_. As the partial density of states (PDOS) of P–Ti_3_C_2_O_2_ and Ti_3_C_2_O_2_ shown (Figure 3c and Figure S4), CB of Ti_3_C_2_O_2_ and P–Ti_3_C_2_O_2_ is mainly contributed by Ti 3d, while the VB originates from the hybridization of Ti 3d, C 2p and O 2p. Moreover, the P atom made a considerable contribution to DOS at the Fermi level, leading to P–Ti_3_C_2_O_2_ owning much higher electronic conductivity.

The migration and diffusion of electrolyte ion H^+^ in electrode materials also affect the electrochemical performance of electrode materials. The calculation results based on the DFT of the free energy for hydrogen atoms diffusion on Ti_3_C_2_O_2_ and P–Ti_3_C_2_O_2_ surfaces in different paths are shown in Figure 3d. (Optimization model structures of H diffusion on the surface of P–Ti_3_C_2_O_2_ and Ti_3_C_2_O_2_ and the detailed diffusion process are shown in Figure S5). Surface H prefers to attach to oxygen atoms on the surface of MXene, so the possible path for H diffusion is chosen between the two nearest O sites. For Ti_3_C_2_O_2_, the surface H directly diffuses from one O site to the nearest O site, that is, from site 1 to 2, and the diffusion free energy is 1.28 eV. For P–Ti_3_C_2_O_2_, we choose two possible paths for the diffusion of H on the surface taking into the effect of P doping, i.e., H diffuses from site Ⅰ to Ⅱ, or from site Ⅱ to Ⅲ. The diffusion free energies for both paths are 0.89 and 0.87 eV, respectively, which is lower than that in Ti_3_C_2_O_2_. The lower diffusion free energy undoubtedly facilitates the diffusion of electrolyte ions on the electrode material surface.

### 2.3. Electrochemical Performances of Electrodes

**Figure 4:**
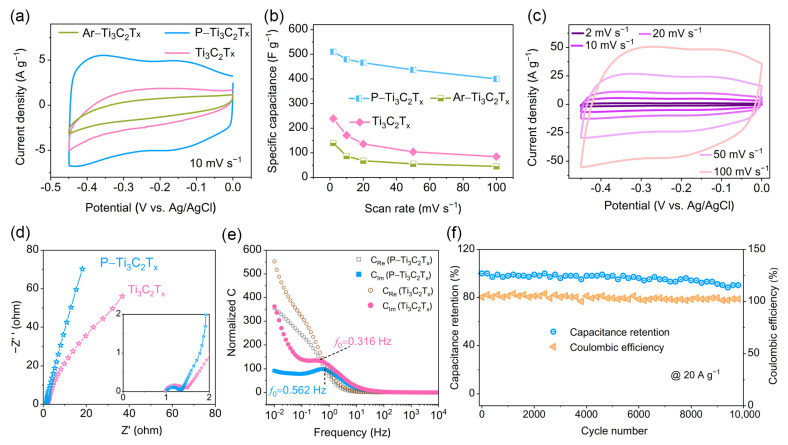
Electrochemical performance. (**a**) CV profiles of Ti_3_C_2_T_x_, P–Ti_3_C_2_T_x_, and Ar–Ti_3_C_2_T_x_; (**b**) Specific capacitance of Ti_3_C_2_T_x_, P–Ti_3_C_2_T_x_, and Ar–Ti_3_C_2_T_x_ calculated by their CV curves; (**c**) CV curves of P–Ti_3_C_2_T_x_ at different scan rate; (**d**) EIS plots of Ti_3_C_2_T_x_ and P–Ti_3_C_2_T_x_; (**e**) Normalized real (C_Re_) and imaginary (C_Im_) parts of capacitance versus frequency of Ti_3_C_2_T_x_ and P–Ti_3_C_2_T_x_; (**f**) Cyclic stability of P–Ti_3_C_2_T_x_ for 10,000 cycles at the current density of 20 A g^−1^.

To verify the practical effect of the change in the interlayer spacing and the introduction of doped P atoms on the electrochemical performance, we first performed CV tests of Ti_3_C_2_T_x_ and P–Ti_3_C_2_T_x_ in 1 M H_2_SO_4_ solution. In Figure 4a, the CV curves of all samples display a rectangular shape, showing capacitive behavior, in which P–Ti_3_C_2_T_x_ has a greater current response. Noticeably, a pair of weak peaks in low voltage (around −0.15 V) appear in the CV curves of the P–Ti_3_C_2_T_x_ electrode, which may be due to the Faraday reaction caused by the doped P atom because it is less electronegative and prefers to share electrons with H; consequently, it can take part in reaction at relatively low voltages. The specific capacitance at different scan rates calculated by CV curves is shown in Figure 4b. The specific capacitance of P–Ti_3_C_2_T_x_ reached 510 F g^−1^ at 2 mV s^−1^, which is higher than that of most reported similar electrode materials, including P–Ti_3_C_2_T_x_, N–doped Ti_3_C_2_T_x_ and N, P–doped graphene (Table S2). At the scan rate of 100 mV s^−1^, the CV curve of P–Ti_3_C_2_T_x_ has no obvious distortion (Figure 4c), and it still delivers a capacitance of 400 F g^−1^ (Figure 4b), capacitance retention maintaining 78% from 2 mV s^−1^ to 100 mV s^−1^, which is higher than 35% of pristine Ti_3_C_2_T_x_. To eliminate the influence of annealing, Ar–Ti_3_C_2_T_x_ was synthesized under the same condition without NaH_2_PO_2_. As shown in Figure S6, Ar–Ti_3_C_2_T_x_ deliveres a minimum electrochemical response area at the same scan rate, that is, it has the smallest specific capacitance, which may be due to the formation of TiO_2_ in the annealing treatment process without NaH_2_PO_2_ with reductive nature.

EIS data can help us investigate the electrical conductivity, charge transfer kinetics, and ion diffusion properties of the electrode. In EIS plots, the high-frequency semicircle diameter is related to the charge transfer resistance (R_ct_) and the diagonal line in the low-frequency region shows charge storage behavior. As shown in Figure 4d and Table S3, P–Ti_3_C_2_T_x_ possesses lower R_ct_ than pure Ti_3_C_2_T_x_ and a higher value of fractional exponent α of constant phase element (CPE), suggesting that P–Ti_3_C_2_T_x_ has a higher electrical conductivity and a more rapid ion diffusion path. This is consistent with the above results for the DOS and H diffusion free energies analysis. Figure 4e shows that P–Ti_3_C_2_T_x_ possesses a smaller characteristic relaxation time constant of τ_0_ (τ_0_ = *f*_0_^−1^), proving the high-rate performance of P–Ti_3_C_2_T_x_ [38,39,40].

For supercapacitors, the long cycle stability of the electrode is one of the key points, because the specific capacitance of the device over time depends on it. Figure 4f exhibits the extended GCD tests approaching 10,000 cycles at a high current density of 20 A g^−1^ for P–Ti_3_C_2_T_x_. Notably, after 10,000 cycles, P–Ti_3_C_2_T_x_ still remains at 90.2% of initial capacitance, which is higher than that of pristine Ti_3_C_2_T_x_ (81.3%, Figure S7) and its Coulombic efficiency of P–Ti_3_C_2_T_x_ is close to 100% in the whole cycles. The enhanced cycle stability may be due to the expanded interlayer space, which allows rapid insertion and removal of electrolyte ions without obvious structure change during the charge–discharge processes.

**Figure 5:**
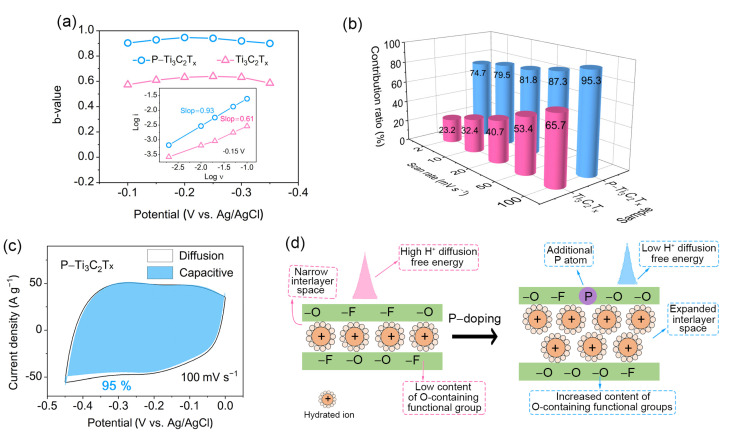
The charge storage mechanism of P–Ti_3_C_2_T_x_ and Ti_3_C_2_T_x_. (**a**) The *b* values of P–Ti_3_C_2_T_x_ and Ti_3_C_2_T_x_; (**b**) Comparison plot between Ti_3_C_2_T_x_ (pink) and P–Ti_3_C_2_T_x_ (blue) of the normalized contribution of capacitive at various scan rates; (**c**) CV data of P–Ti_3_C_2_T_x_ corresponding to the capacitive contribution for the total current at 100 mV s^−1^; (**d**) Schematic illustration of charge storage of hydrated electrolyte ions in Ti_3_C_2_T_x_ and P–Ti_3_C_2_T_x_.

To gain insight into the charge storage mechanism of the electrodes, the surface capacitive contribution and the diffusion control contribution are analyzed. First, we plotted the log *i*-log *v* curve, using Formula [41]:

log i=b·log v+log a

where *i* and *v* present currents under various voltage and scan rates of CV curves, respectively. *b* and *a* present slope of the log *i*-log *v* plot and constant. Figure 5a displays the *b*-values of P–Ti_3_C_2_T_x_ and Ti_3_C_2_T_x_ as calculated at different voltages corresponding to the CV curves. It is clear that P–Ti_3_C_2_T_x_ owns higher *b* values (closed to 1) than pure Ti_3_C_2_T_x_ (closed to 0.5), indicating P–Ti_3_C_2_T_x_ shows a surface-controlled charge storage behavior in the electrochemical processes whereas the electrochemical processes of pristine Ti_3_C_2_T_x_ are dominated by diffusion-controlled charge storage behavior. More specifically, the contribution proportion of the two mechanisms in total capacitance can be quantitatively distinguished at a randomizing scan rate according to the following Formula [42]:

$$i\;\left(V\right)=k_{1}\cdot v+k_{2}\cdot v^{1/2}$$

where i (V) corresponds to the current at the specific voltage, v stands for the scan rate, $k_{1}\cdot v$ and $k_{2}\cdot v^{1/2}$ represent the surface capacitive control and the diffusion control, respectively. The results show that the proportion of surface capacitive contribution increases with the increase in scan rates for both electrodes (Figure 5b,c, Figures S8 and S9). Notably, the proportion of the surface capacitive contribution of P–Ti_3_C_2_T_x_ is always larger than that of pure Ti_3_C_2_T_x_ at various scan rates, which is consistent with the *b*-value analysis results. The high surface capacitance contribution implies that there is a fast ion diffusion in P–Ti_3_C_2_T_x_, which is mainly due to the expanded interlayer spacing and lower diffusion free energy in P–Ti_3_C_2_T_x_. We can also understand the electrochemical process of P–Ti_3_C_2_T_x_ MXene from the in situ electrochemical EIS results. Figure S10 shows the in situ EIS diagram during the discharge process. In the low-frequency range of Nyquist plots around open circuit potential (−0.02 V), the curve is characterized by a diagonal. When the voltage changes from −0.02 V to −0.06 V, the imaginary part of the impedance rises sharply (almost vertically), indicating that the inserted hydrated cation increases the interlayer spacing, resulting in fast and free ion transport. As the discharge reaction proceeds, the diagonals of the EIS plots are always approximately perpendicular, indicating a uniform and expanded interlayer space exists in P–Ti_3_C_2_T_x_, which improves ion accessibility to the active site, resulting in high specific energy storage capacity. Here, the decrease in impedance imaginary part value from −0.06 V to −0.25 V should be attributed to the continuous accumulation of charge.

As discussed above, the enhanced electrochemical performance should be ascribed to several reasons as follows (Figure 5d). The expansion of interlayer spacing facilitates rapid H^+^ transport. The increased content of O-containing functional groups increases the hydrophilicity of the electrode, which is conducive to the contact of MXene surface with the electrolyte and thus increases active site accessibility. The increase in O-containing functional groups provides more active sites and the doped P atoms bring additional active sites, which greatly increase the specific capacitance. The change in surface functional groups causes the increase in Ti content of the high valence state with an electrochemical activity that can accommodate more electrons during discharge. Moreover, the introduction of P increases the electrical conductivity of MXene and reduces the free energy of H^+^ diffusion on the MXene surface, which is conducive to electrochemical reactions.

## 3. Experiment

### 3.1. Synthesis of Pristine Ti_3_C_2_T_x_ MXene

The Ti_3_C_2_T_x_ MXene was prepared by etching Al layer from Ti_3_AlC_2_ MAX phase which was prepared in the house laboratory by the solid−liquid reaction synthesis method [43] (Figures S1 and S2). In detail, 3 g Ti_3_AlC_2_ powder was slowly added into 60 mL etching solution (9 M HCl, 12 M LiF) at room temperature for 20 days to remove the Al layer. The suspension was rained by vacuum filtration and the sediment was washed with 1 M HCl followed by deionized water until the pH reached 6. Finally, the obtained product was dried at 60 °C overnight to produce Ti_3_C_2_T_x_ powder.

### 3.2. Synthesis of P–Ti_3_C_2_T_x_ and Ar–Ti_3_C_2_T_x_

The P–Ti_3_C_2_T_x_ was fabricated by annealing a mixture of Ti_3_C_2_T_x_ powder and NaH_2_PO_2_∙H_2_O. In brief, 200 mg Ti_3_C_2_T_x_ powder was mixed with 20 mL DI water to form a suspension under ultra-sonic treatment for 5 min, and then 2 g NaH_2_PO_2_·H_2_O was dissolved in the above suspension. Then, the mixed solution was freeze-dried, placed in a tube furnace, and annealed under Ar flow at 400 °C for 2 h with a heating rate of 2 °C min^−1^. Finally, the resulting product was rinsed with DI water to remove the resultant phosphate salts. Ar–Ti_3_C_2_T_x_ was prepared under the same condition without NaH_2_PO_2_∙H_2_O.

### 3.3. Material Characterizations

The microstructure morphology and element distribution were characterized using a JEOL JSM-6700F (JEOL Japan Electronics Co., Ltd., Tokyo, Japan) field emission FESEM equipped with EDS equipment. More detailed structural information was acquired by using a JEM-2100PLUS (JEOL Japan Electronics Co., Ltd.) TEM equipment. The FTIR spectroscopy was performed on a Nicolet IS10 (Thermo Fisher Scientific, Waltham, MA, USA) device. The XRD pattern of the samples was recorded using a Rigaku MiniFlex (Rigaku Corporation, Tokyo, Japan) X-ray diffractometer. N_2_ adsorption–desorption tests were performed using Micromeritics ASAP2460-2 (Micromeritics Instruments Corporation, Norcross, GA, USA). The XPS spectra analysis was performed to further characterize the chemical compositions on a Thermo ESCALAB Xi^+^ (Thermo Fisher Scientific (China) Co., Ltd., Shanghai, China) device with an Al-Ka excitation source.

### 3.4. Computational Detail

Density functional theory (DFT) was performed by Vienna Ab-initio Simulation Package (VASP) software [44] with the projector augmented wave (PAW) method [45]. The convergence threshold of energy was set as 10^−5^ eV and the convergence threshold of maximum stress was 0.05 eV/Å. The cut-off energy was set as 400 eV, and the k-points were 2 × 2 × 1. Hubbard U correction was set, and the U value was 4.0 eV for 3d orbit Ti.

### 3.5. Electrochemical Measurements

Electrochemical performance tests were carried out in a three-electrode system on an electrochemical workstation (CHI 660E, Shanghai Chenhua Instrument Co., Shanghai, China) at room temperature, where Ag/AgCl, Pt wire, and 1 M H_2_SO_4_ solution as reference electrode, counter electrode, and electrolyte, respectively. For working electrode, a mixture of 80 wt% active material, 10 wt% polyvinylidene fluoride, and 10 wt% carbon black in N-methyl pyrrolidone was ground in an agate mortar until the slurry was grain-free, and then coated on copper foil and dried at 60 °C for 12 h. The electrochemical performances were studied by cyclic voltammetry (CV) and electrochemical impedance spectroscopy (EIS) techniques.

## 4. Conclusions

In summary, P-doped Ti_3_C_2_T_x_ MXene was prepared by a simple annealing method with NaH_2_PO_2_ as phosphorus source, which delivers a high specific capacitance of 510 F g^−1^ and 400 F g^−1^ at the scan rate of 2 mV s^−1^ and 100 mV s^−1^. Morphology and component characterization, DFT calculation, and electrochemical kinetic analysis have been used to give deep insight into the mechanism of improvement in electrochemical performance. The expansion of interlayer spacing facilitates rapid H^+^ transport. The increased content of O-containing functional groups and the introduction of P atoms not only increase the hydrophilicity of the electrode material but also bring more active sites. The change in surface functional groups causes the increase in Ti content of the high valence state with a higher electrochemical activity that can accommodate more electrons during discharge. Moreover, the introduction of P improves the electrical conductivity of MXene and reduces the free energy of H^+^ diffusion on the MXene surface. Synergistic modification of interlayer structure and chemical state improves the possibility of Ti_3_C_2_T_x_ for accommodating more H^+^ ions. This work opens avenues for the development of other 2D materials in constructing high-energy storage systems by heterogeneous atom doping. 

## Data Availability

Not applicable.

## Supplementary Materials

The following supporting information can be downloaded at: https://www.mdpi.com/article/10.3390/molecules28134892/s1, Figure S1: The Ti_3_C_2_T_x_ MXene was prepared from Ti_3_AlC_2_ MAX phase by etching Al layer; Figure S2: (a) SEM image and (b) XRD pattern of Ti_3_AlC_2_ MAX phase particles; Figure S3: XRD pattern of Ti_3_C_2_T_x_, Ar–Ti_3_C_2_T_x_ and P–Ti_3_C_2_T_x_; Figure S4: PDOS of Ti_3_C_2_T_x_; Figure S5: Optimization model structures of H diffusion on the surface of (a) P–Ti_3_C_2_O_2_ and (b) Ti_3_C_2_O_2_ and the detailed diffusion process; Figure S6: CV profiles of (a) Ti_3_C_2_T_x_ and (b) Ar–Ti_3_C_2_T_x_. The area of CV curves of both samples increases with the increase in scan rate; Figure S7: Cyclic stability of Ti_3_C_2_T_x_ for 10,000 cycles at the current density of 20 A g^−1^; Figure S8: Kinetic and quantitative analysis of the P–Ti_3_C_2_T_x_ electrode; Figure S9: Kinetic and quantitative analysis of the Ti_3_C_2_T_x_ electrode; Figure S10: In situ EIS spectra of P–Ti_3_C_2_T_x_ electrodes; Table S1: BET specific surface area of the electrode materials; Table S2: Comparison of electrochemical performance between P–Ti_3_C_2_T_x_ and previously reported heteroatom-doped MXene electrodes [26,27,28,31,37,46]; Table S3: EIS fitting results of Ti_3_C_2_T_x_ and P–Ti_3_C_2_T_x_.

## Safety Statement

As for the synthesis of P–Ti_3_C_2_T_x_, the toxic gas PH_3_ is released during the heat treatment process. Therefore, an exhaust gas treatment device must be equipped.
