# Supplementary material for: Phosphorus Doping Strategy-Induced Synergistic Modification of Interlayer Structure and Chemical State in Ti3C2Tx toward Enhancing Capacitance
Source: Molecules. 2023 Jun 21;28(13):4892. doi: 10.3390/molecules28134892 (PMC10343897; doi:10.3390/molecules28134892)
Supplement: Supplementary file 1 [file molecules-28-04892-s001.zip › molecules-2440710-supplementary.pdf]

Supplementary Materials

Phosphorus Doping Strategy-Induced Synergistic Modification of Interlayer Structure and Chemical State in $Ti_3C_2T_x$ toward Enhancing Capacitance

Lihong Chen [1], Yifan Bi [1], Yunqi Jing [2], Jun Dai [3], Zhenjiang Li [1], Changlong Sun [1], Alan Meng [4], Haijiao Xie [5] and Minmin Hu [1,*]

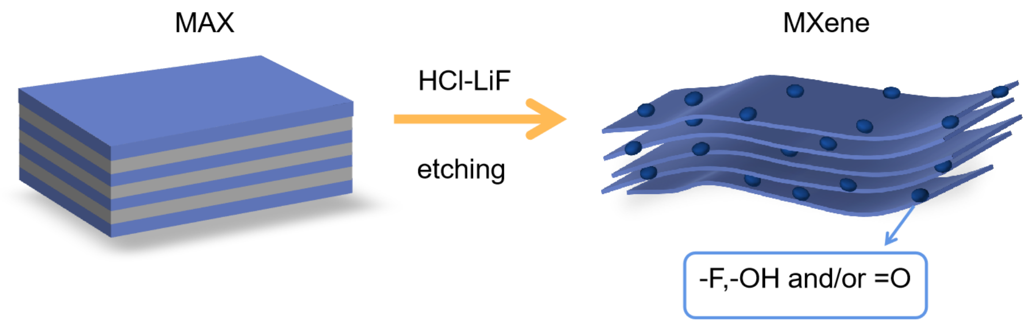

Figure S1. The $Ti_3C_2T_x$ MXene was prepared from Ti_3AlC_2 MAX phase by etching Al layer. The surface of etched $Ti_3C_2T_x$ is terminated with functional groups, such as –F, –OH and =O.

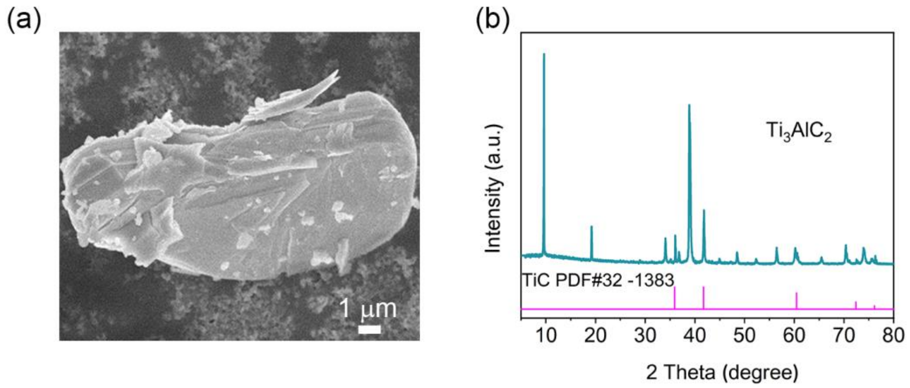

Figure S2. (**a**) SEM image and (**b**) XRD pattern of Ti_3AlC_2 MAX phase particles.

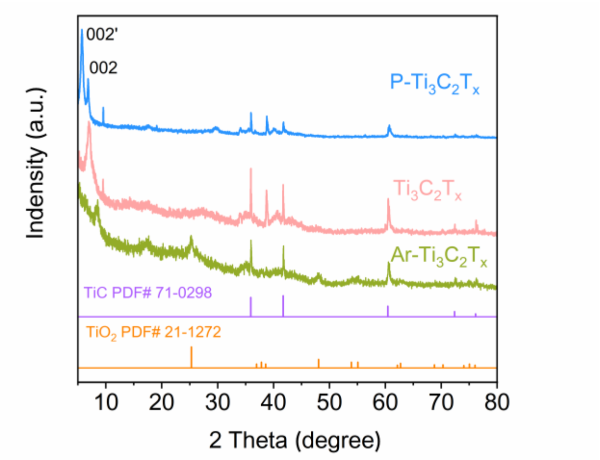

Figure S3. XRD pattern of $Ti_3C_2T_x$, Ar–$Ti_3C_2T_x$ and P–$Ti_3C_2T_x$. (002) peak of P–$Ti_3C_2T_x$ shifts to a lower angel and splits into two peaks, while Ar–$Ti_3C_2T_x$ moves to a higher angel and the peaks of TiO_2 appear.

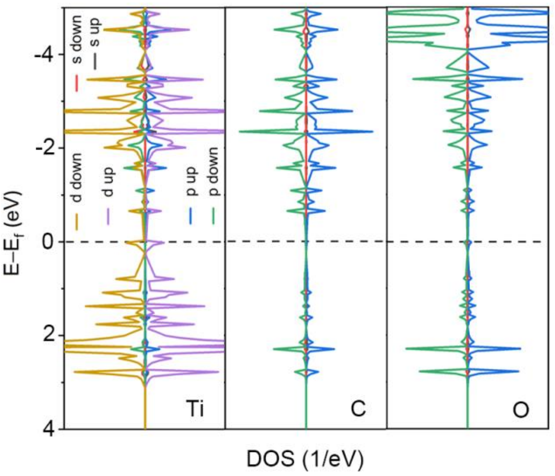

Figure S4. PDOS of $Ti_3C_2O_2$.

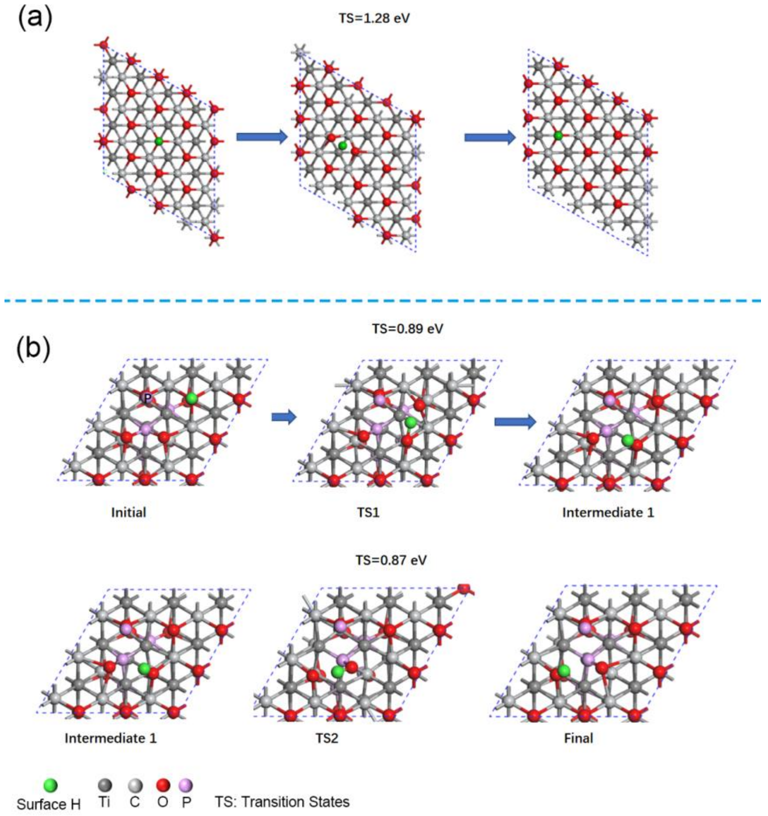

Figure S5. Optimization model structures of H diffusion on the surface of (a) P–$Ti_3C_2O_2$ and (b) $Ti_3C_2O_2$ and the detailed diffusion process

.

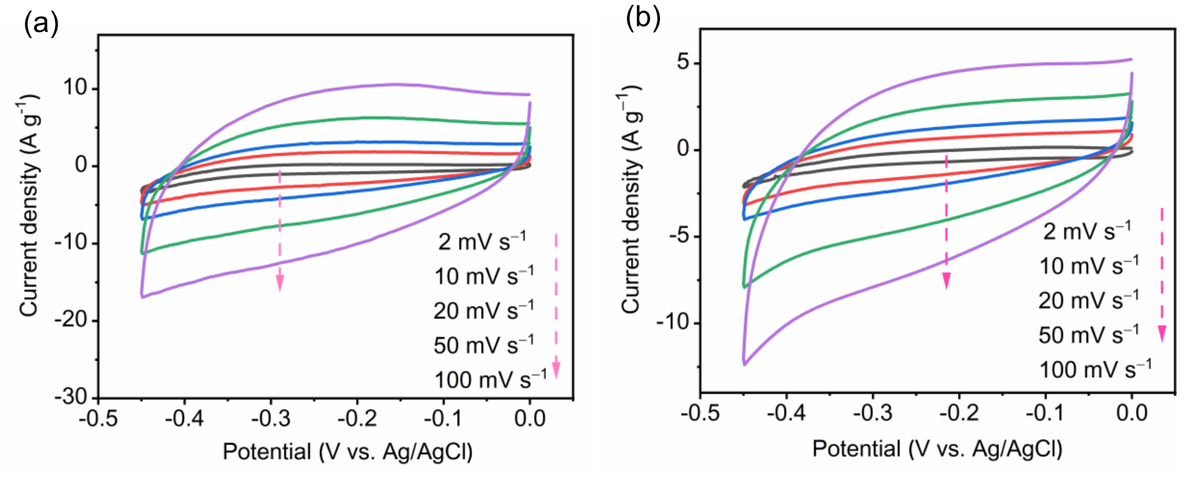

Figure S6. CV profiles of (**a**) $Ti_3C_2T_x$ and (**b**)Ar–$Ti_3C_2T_x$. The area of CV curves of both samples increases with the increase in scan rate.

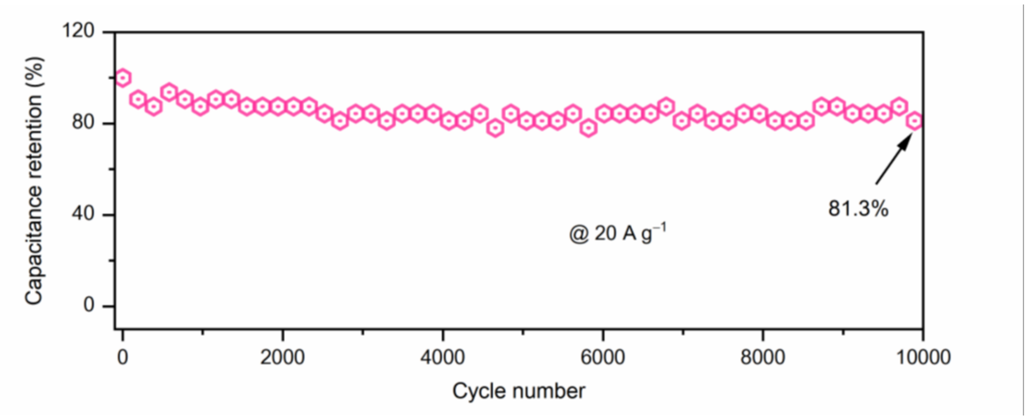

Figure S7. Cyclic stability of $Ti_3C_2T_x$ for 10, 000 cycles at the current density of 20 A g^{-1}.

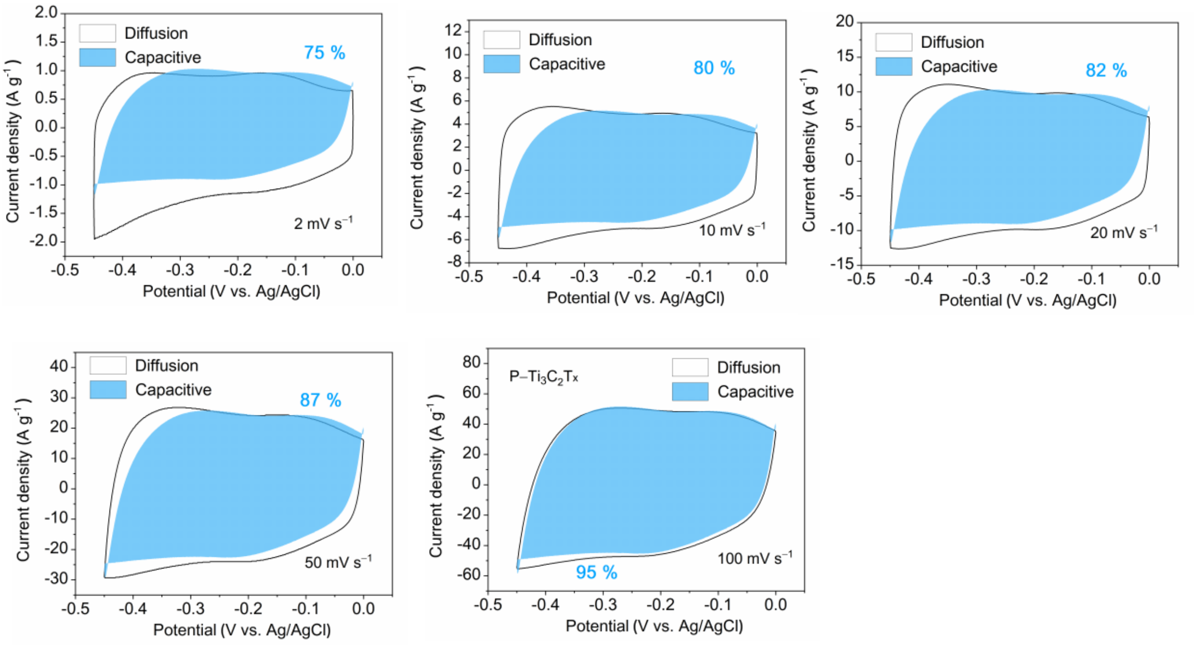

Figure S8. Kinetic and quantitative analysis of the P–$Ti_3C_2T_x$ electrode. Capacitive (blue) and diffusion-controlled (white) contribution at 2, 10, 20, 50 and 100 mV s^{-1}.

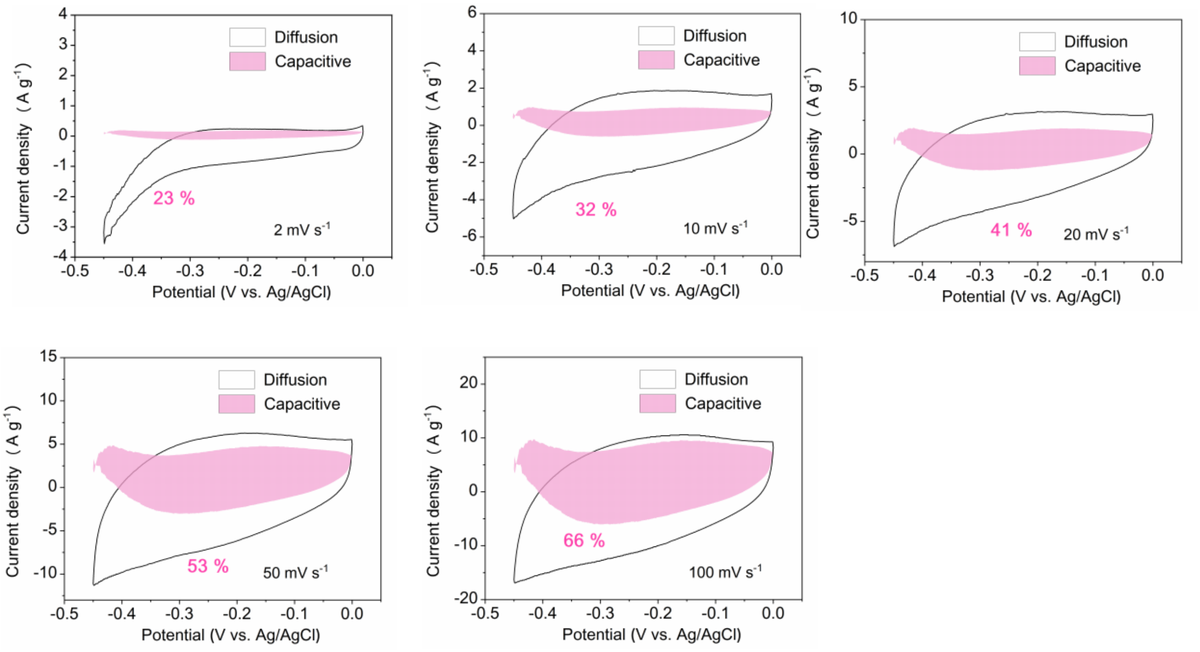

Figure S9. Kinetic and quantitative analysis of the $Ti_3C_2T_x$ electrode. Capacitive (pink) and diffusion-controlled (white) contribution at 2, 10, 20, 50 and 100 mV s^{-1}.

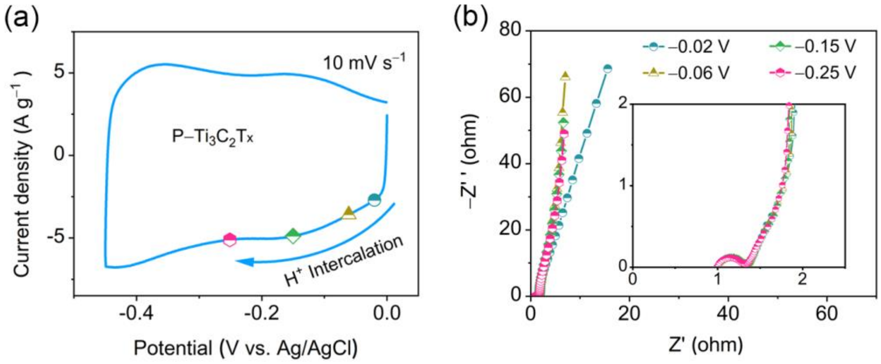

Figure S10. In situ EIS spectra of P−$Ti_3C_2T_x$ electrodes. (**a**) CV profile; (**b**) EIS data collected at different potentials vs. Ag/AgCl on the intercalation branches for P−$Ti_3C_2T_x$ electrodes.

Table S1. BET specific surface area of the prepared electrode materials.

Samples	S_{BET} ($m^2 g^{-1}$)
$Ti_3C_2T_x$	5.0871
P–$Ti_3C_2T_x$	5.3403

Table S2. Comparison of electrochemical performance between P–$Ti_3C_2T_x$ and previously reported heteroatom-doped MXene electrodes.

Electrode material	Electrolyte	Specific Capacitance	Reference
**P–$Ti_3C_2T_x$**	**1 M H_2SO_4**	**520 F g^{-1} at 2 mV s^{-1}**	**This work**
P–$Ti_3C_2T_x$	1 M H_2SO_4	320 F g^{-1} at 0.5 A g^{-1}	[1]
P–$Ti_3C_2T_x$	3 M H_2SO_4	448 F g^{-1} at 1 A g^{-1}	[2]
N,P–G	1 M H_2SO_4	183 F g^{-1} at 0.05 A g^{-1}	[3]
N–$Ti_3C_2T_x$	1 M H_2SO_4	192 F g^{-1} at 1 mV s^{-1}	[4]
N–$Ti_3C_2T_x$ film	1 M H_2SO_4	340 F g^{-1} at 2 mV s^{-1}	[5]
S,N-MXene/rGO	1 M H_2SO_4	246.9 F g^{-1} at 1 A g^{-1}	[6]

Table S3. EIS fitting results of $Ti_3C_2T_x$ and P–$Ti_3C_2T_x$

Element	$Ti_3C_2T_x$ (Error%)	P–$Ti_3C_2T_x$ (Error%)
Rs	1.03 (1.565%)	1.067 (1.097%)
C	0.00018098 (11.046%)	0.00022255 (11.265%)
R_{ct}	0.42459 (4.3362%)	0.28853 (4.3645%)
CPE-P	0.058259 (2.1657%)	0.10428 (1.187%)
CPE-α	0.75446 (0.55873%)	0.85061 (0.33992%)

References

1. Wen, Y.; Li, R.; Liu, J.; Wei, Z.; Li, S.; Du, L.; Zu, K.; Li, Z.; Pan, Y.; Hu, H. A temperature-dependent phosphorus doping on $Ti_3C_2T_x$ MXene for enhanced supercapacitance. *J. Colloid Interface Sci.* **2021**, *604*, 239-247.
2. Gupta, N.; Sahu, R. K.; Mishra, T.; Bhattacharya, P. Microwave-assisted rapid synthesis of titanium phosphate free phosphorus doped Ti_3C_2 MXene with boosted pseudocapacitance. *J. Mater. Chem. A* **2022**, *10*, 15794-15810.
3. Wen, Y.; Rufford, T. E.; Hulicova-Jurcakova, D.; Wang, L. Nitrogen and phosphorous co-doped graphene monolith for supercapacitors. *ChemSusChem* **2016**, *9*, 513-520.
4. Wen, Y.; Rufford, T. E.; Chen, X.; Li, N.; Lyu, M.; Dai, L.; Wang, L. Nitrogen-doped $Ti_3C_2T_x$ MXene electrodes for high-performance supercapacitors. *Nano Energy* **2017**, *38*, 368-376.
5. Zhang, T.; Xiao, J.; Li, L.; Zhao, J.; Gao, H. A high-performance supercapacitor electrode based on freestanding N-doped $Ti_3C_2T_x$ film. *Ceram. Int.* **2020**, *46*, 21482-21488.
6. Liao, L.; Jiang, D.; Zheng, K.; Zhang, M.; Liu, J. Industry-scale and environmentally stable $Ti_3C_2T_x$ MXene based film for flexible energy storage devices. *Adv. Func. Mater.* **2021**, *31*, 2103960.
